# Concurrent Olaparib and Radiation Therapy for *BRCA2*-Mutated Breast Cancer

**DOI:** 10.1016/j.adro.2024.101528

**Published:** 2024-04-27

**Authors:** Danny Lavigne, Lucas Sideris, Lara de Guerke, Eve-Lyne Marchand, Suzanne Fortin, Pierre Dubé, Peter Vavassis, Marie-Hélène Auclair, Michael Yassa

**Affiliations:** a Department of Radiation Oncology, Hôpital Maisonneuve-Rosemont, University of Montreal, Montreal, Quebec, Canada; b Department of Surgery, Hôpital Maisonneuve-Rosemont, University of Montreal, Montreal, Quebec, Canada; c Department of Gynecology, Hôpital Maisonneuve-Rosemont, University of Montreal, Montreal, Quebec, Canada

## Introduction

Pathogenic mutations in the *BRCA1* or *BRCA2* genes lead to genomic instability through a defective homologous recombination pathway, an essential repair mechanism for DNA double-strand breaks.^1^ Poly (adenosine diphosphate-ribose) polymerase inhibitors such as olaparib take advantage of this defect by indirectly increasing the amount of unrepaired DNA double-strand breaks in *BRCA1/2*-mutated cancer cells through inhibition of the base excision repair pathway, ultimately leading to cell death.^2^ Although adjuvant olaparib has been shown to improve survival in patients with *BRCA1/2*-mutated breast cancer, there is a paucity of evidence regarding its safety when combined with radiation therapy, which generates a significant amount of DNA damage.^3^^,^^4^ We hereby present the acute toxicities of locoregional breast radiation therapy with concurrent olaparib in a patient treated for *BRCA2*-mutated breast cancer.

## Case Description

### Patient history and presentation

A 45-year-old woman was initially treated for triple-negative, multifocal, clinically T2N0M0 invasive ductal carcinoma of the left breast, according to the 8th edition of the American Joint Committee on Cancer's staging system.^5^ She received neoadjuvant chemotherapy followed by bilateral total mastectomy and immediate reconstruction with breast implants as well as a left axillary sentinel lymph node biopsy in June 2019, revealing a pathologic complete response. No adjuvant radiation therapy was administered. She presented a biopsy-proven locoregional recurrence in January 2022 with a palpable nodularity in her left tail of Spence. A fluorodeoxyglucose-positron emission tomography documented a 2-cm lesion superior and lateral to the left breast implant along with a 1-cm left axillary lymph node. The fluorodeoxyglucose-positron emission tomography also revealed bilateral ovarian lesions with multiple peritoneal carcinomatosis implants, but no distant visceral or bone metastases. A diagnostic laparoscopy confirmed a synchronous Fédération Internationale de Gynécologie et d'Obstétrique stage IIIC high-grade serous ovarian cancer.^6^ A germline genetic analysis was performed by next-generation sequencing and deletion/duplication testing of 22 cancer predisposition genes (*APC, ATM, BMPR1A, BRCA1, BRCA2, BRIP1, CDH1, CHEK2, EPCAM, MLH1, MSH2, MSH6, MUTYH, NBN, PALB2, PMS2, PTEN, RAD51C, RAD51D, SMAD4, STK11*, and *TP53*) using an Invitae multigene panel. Genomic DNA obtained from a blood sample was enriched for targeted regions and sequenced using Illumina technology (Illumina, Inc). The analysis revealed a germline pathogenic *BRCA2* mutation (heterozygous c.6468_6469del [p.Gln2157Ilefs*18] mutation in exon 11).

### Management

**Figure 1:**
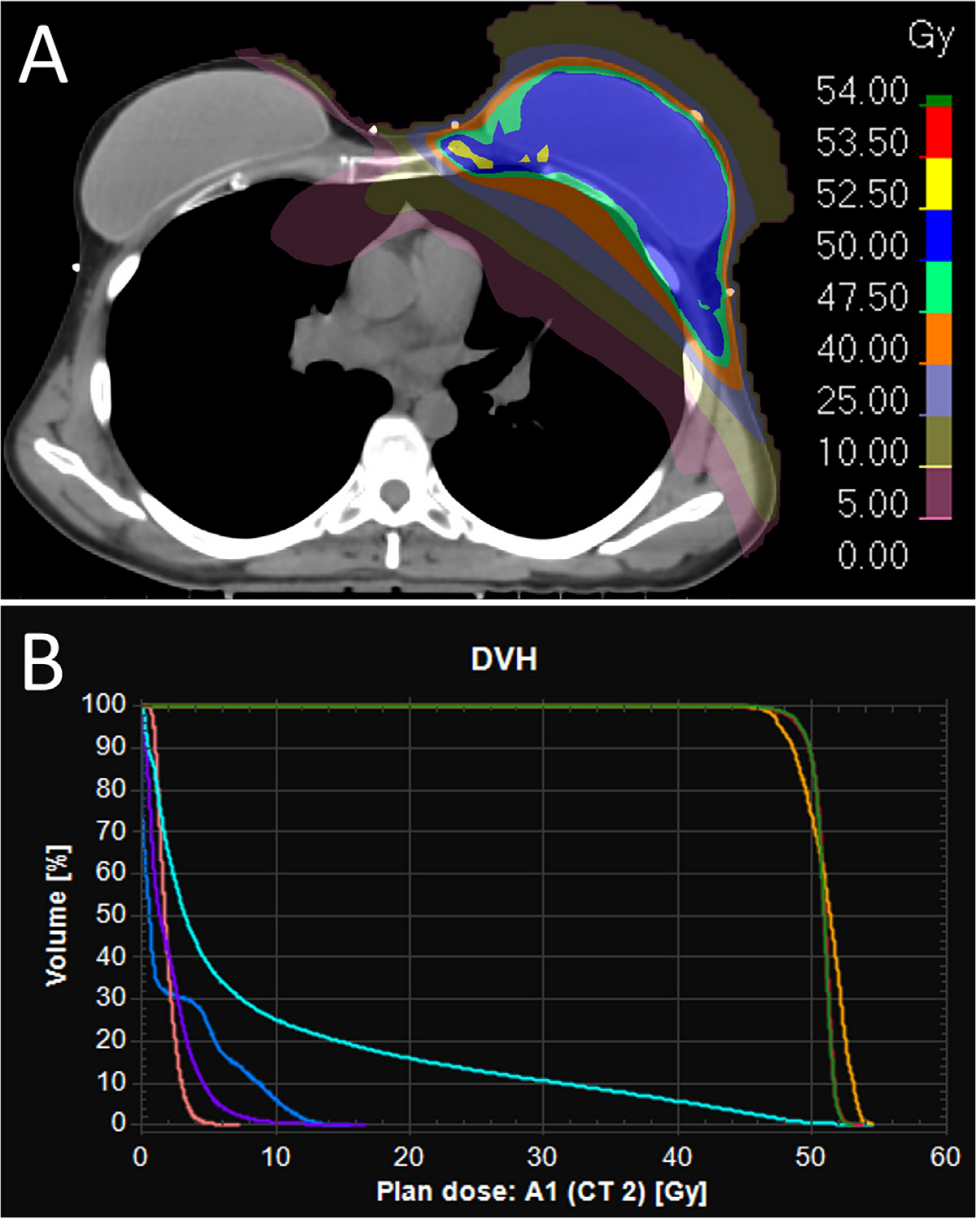
Radiation dose distribution on an axial computed tomography slice at the level of the reconstructed breast (A) and dose-volume histogram (B) of the presented patient's radiation therapy plan, the latter representing the volumetric radiation dose received by the breast planning target volume (red curve), axillary and supraclavicular planning target volume (green curve), internal mammary chain planned target volume (orange curve), left lung (cyan curve), spinal cord (blue curve), right lung (purple curve), and heart (pink curve).

All procedures described in the present study were performed in accordance with institutional review board-approved protocols and were approved by multidisciplinary tumor boards. The patient underwent a complete surgical cytoreduction for her ovarian cancer followed by adjuvant chemotherapy with 6 cycles of carboplatin and paclitaxel from May to August 2022, which simultaneously served as neoadjuvant chemotherapy for her synchronous breast cancer. She subsequently underwent a left-sided lumpectomy with a sentinel lymph node biopsy and targeted axillary dissection in September 2022, which revealed a residual 2.5-mm, grade 2, triple-negative invasive ductal carcinoma, without lymphovascular invasion, resected with clear surgical margins, and 1 out of 10 lymph nodes harboring a 3-mm metastasis. She began an adjuvant treatment of oral olaparib at the dose of 300 mg twice daily in October 2022. After consultation with a radiation oncologist and presentation of the patient's case at the institutional breast and gynecologic multidisciplinary tumor boards and based on a recently published phase 1 study,^7^ an adjuvant treatment of radiation therapy for her breast cancer with concomitant olaparib at a reduced dose of 200 mg twice daily was favored. The patient agreed to the proposed treatment after discussing the potential benefits, risks, and alternative therapeutic options. She received 50 Gy of radiation therapy to the left reconstructed breast, axillary levels I-III, supraclavicular and internal mammary chain nodal regions in 25 daily doses of 2 Gy with a deep inspiration breath hold technique, delivered with volumetric arc therapy, from December 13, 2022, to January 18, 2023. The dose distribution and dose-volume histogram of the treatment plan are shown in Fig. 1. Olaparib was reduced from 300 mg twice daily to 200 mg twice daily 1 week before the start of radiation therapy, and the initial dose was resumed 1 day after the end of treatments.

### Follow-up

**Figure 2:**
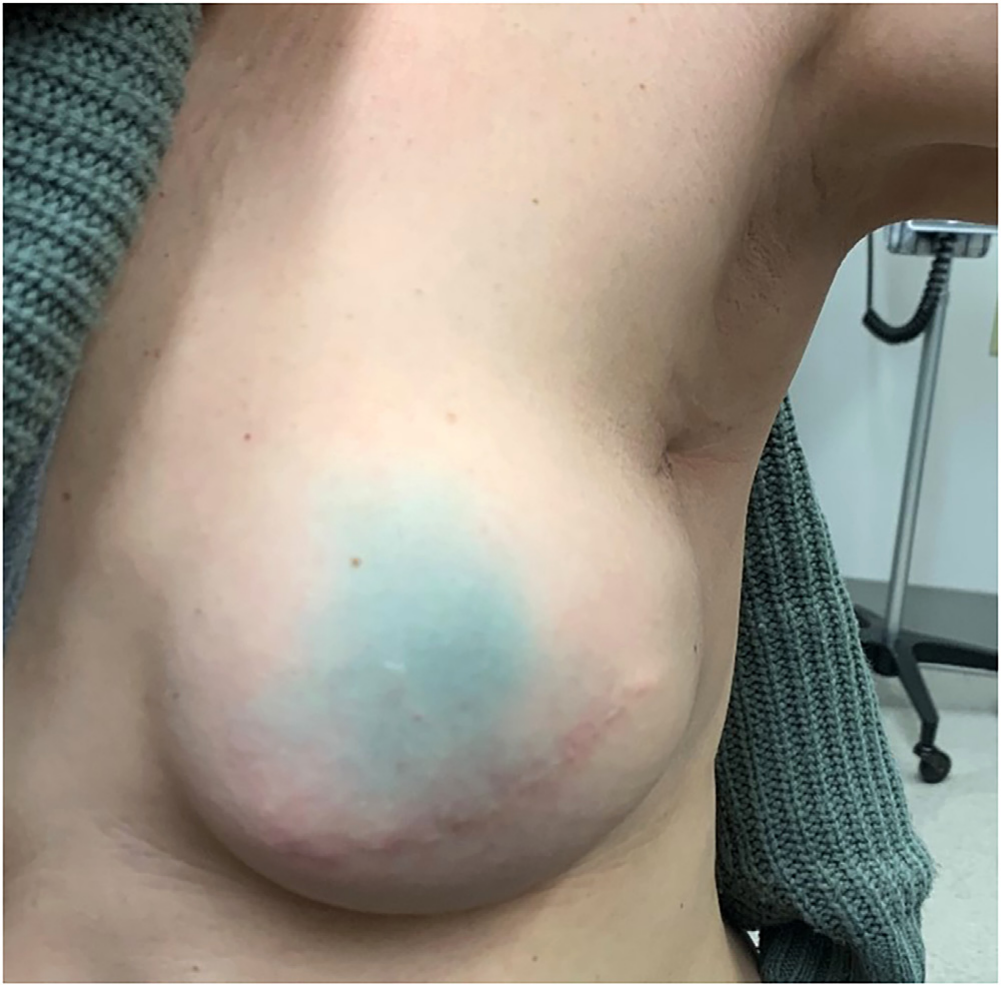
The presented patient's cosmetic results 6 weeks after her course of concurrent olaparib and radiation therapy, showing complete resolution of her mild radiodermatitis with no residual treatment sequelae. The temporary blue coloration of her left breast is due to the patent blue dye used for sentinel lymph node biopsy.

The patient was assessed after 16 Gy of radiation and had developed faint erythema of the left breast and axillary fold with mild odynophagia, for which a nonmedicinal hydrating cream and an oral solution of diphenhydramine, nystatin, and hydrocortisone were prescribed. She was reassessed after 40 Gy, at which point her odynophagia had resolved. On her last day of radiation therapy, she remained with a faint erythema of the treated region, which was slightly more pronounced in the axillary fold, but with no moist desquamation. Routine blood work the following week revealed a lymphopenia measured at 0.30 × 10^9^ lymphocytes/L, compared with a normal value of 1.50 × 10^9^ lymphocytes/L 3 weeks before radiation therapy. No signs or symptoms of an active infectious process were noted. At her 6-week follow-up, the erythema had completely resolved, and she did not report any breast or chest wall pain or respiratory symptoms. The physical examination of the breast and axilla were unremarkable, with no erythema, induration, or palpable axillary lymph node. The patient's 6-week cosmetic results are shown in Fig. 2.

All data included in this case report were retrospectively collected from the patient's electronic medical record. The current study was conducted according to the Declaration of Helsinki. The patient provided written informed consent for the publication of her case, including the publication of potentially identifiable images. All procedures described in the present study were performed in accordance with institutional review board-approved protocols at Hôpital Maisonneuve-Rosemont and were approved by multidisciplinary tumor boards.

## Discussion

The combination of olaparib and radiation therapy has shown promising results in preclinical studies, with evidence suggesting that olaparib may enhance the radiosensitivity of breast cancer cells, potentially leading to improved treatment outcomes.^8^ However, clinical data regarding the safety of combining these 2 treatment modalities with potentially overlapping biologic mechanisms are lacking. To our knowledge, only 1 clinical study investigated this combined treatment in patients with breast cancer. The phase I dose-escalation trial by Loap et al^7^ investigated a maximum tolerated dose of olaparib with concurrent radiation therapy in 24 patients, with doses ranging from 50 to 200 mg twice daily. A radiation dose of 50 to 50.4 Gy in 25 to 28 fractions was delivered either to the whole breast or the chest wall, with a simultaneously integrated boost up to 63 Gy to the tumor bed in patients younger than 60 years undergoing breast-conserving surgery. The authors reported no dose-limiting toxicity, no late treatment-related grade 3 or greater adverse events, and only 1 patient suffered from chronic grade 2 breast pain, fibrosis, and deformity. In the acute setting, they reported 2 grade 3 radiodermatitis, 1 grade 3 breast pain, 1 grade 3 lymphocele, 8 grade 3 lymphopenias, and 3 grade 4 lymphopenias. Of note, only 5 patients received the maximum dose of 200 mg twice daily, only 7 were known for a germline *BRCA1/2* mutation, and only 14 received whole-breast radiation therapy, and the remainder received postmastectomy irradiation. The authors concluded that, at these doses, breast radiation therapy with concomitant olaparib is well tolerated and should be further studied. The phase III OlympiA trial, which demonstrated increased survival with olaparib for patients with early-stage *BRCA1/2*-mutated breast cancer, administered olaparib after the completion of adjuvant radiation therapy, thus providing no information on the safety of concurrent treatment.^3^^,^^4^

The present case report therefore incorporates reassuring data to the very limited body of evidence surrounding the safety of concomitant olaparib and radiation therapy in patients with germline *BRCA1/2* pathogenic mutations. Despite receiving concurrent olaparib, the presented patient did not experience any unusual clinical acute toxicities from postmastectomy and regional nodal irradiation, which were limited to mild dermatitis and esophagitis. This is particularly reassuring in the context of the potentially overlapping biologic mechanisms of these 2 treatment modalities. As radiation therapy induces a multitude of DNA damage, including base mutations and double-strand breaks, it could be predicted that concurrent olaparib, which inhibits the base excision repair pathway, would lead to excessive cell death and toxicity in the treatment field.^9^ Considering the safety results reported by Loap et al^7^ and corroborated by our presented case, it could be postulated that the other DNA repair pathways may sufficiently repair the increased damage in normal tissues to prevent excess toxicity. Although currently theoretical, this would further widen the therapeutic index by selectively increasing the radiosensitivity of *BRCA1/2*-mutated cancer cells, which consistently harbor biallelic function loss and consequent homologous recombination deficiency.^8^^,^^10^ Although it did not translate to any clinical complication, the patient did develop a grade 3 lymphopenia according to the Common Terminology Criteria for Adverse Events (version 5.0), consistent with the high frequency of lymphopenia reported by Loap et al.^7^^,^^11^ This toxicity was likely related to the combination of olaparib and radiation therapy, because it only appeared after the concurrent treatment, and it may warrant a closer monitoring of blood work in patients undergoing this treatment. It must be noted that the patient described in this report received radiation therapy to a reconstructed breast after a previous mastectomy, thus limiting the extrapolation of her results to patients treated after a breast-conserving surgery. However, as previously noted, 14 of the 24 patients included in the study by Loap et al^7^ received whole-breast radiation therapy after a partial mastectomy.

The SOLO1 trial demonstrated a significant improvement in median progression-free survival and a trend toward better overall survival with maintenance olaparib for patients known to have a *BRCA* mutation treated for advanced ovarian cancer.^12^^,^^13^ The main concern with temporarily ceasing olaparib during radiation therapy for the presented patient was thus the risk of jeopardizing the control of her synchronous advanced ovarian cancer. Furthermore, it is notably important to assess the safety of this concurrent approach because radiation therapy is indicated for most women with early-stage breast cancer eligible for olaparib. Increasing the body of evidence regarding the safety of this concurrent treatment could allow a prompt and uninterrupted treatment with olaparib in such patients presenting with high-risk disease. To our knowledge, there is only 1 ongoing phase II clinical trial currently assessing this treatment combination for patients with breast cancer, although with a lower dose of olaparib of only 25 mg twice daily.^14^ In the era of precision medicine, the presented patient's situation highlights the need for more data regarding this concurrent approach, which could potentially maximize outcomes of both her breast and ovarian cancers.

In conclusion, clinical data regarding the safety of concurrent olaparib and radiation therapy in patients with *BRCA1/2*-mutated breast cancer are very limited. This case report suggests that this treatment approach is well tolerated in the acute setting, with no increased or unexpected toxicity. Further clinical trials are needed to confirm the safety and efficacy of concurrent olaparib and radiation therapy in this patient population.

## Disclosures

Eve-Lyne Marchand reports a relationship with AFX Medical that includes consulting or advisory. Peter Vavassis reports a relationship with AbbVie that includes board membership, consulting or advisory, and speaking and lecture fees. Peter Vavassis reports a relationship with Bayer that includes board membership and consulting or advisory. Peter Vavassis reports a relationship with Boston Scientific that includes board membership and consulting or advisory. Peter Vavassis reports a relationship with Knight Therapeutics that includes board membership and consulting or advisory. Peter Vavassis reports a relationship with Novartis that includes board membership and consulting or advisory. Peter Vavassis reports a relationship with Sanofi that includes board membership and consulting or advisory. Peter Vavassis reports a relationship with Sumitomo Pharma that includes board membership and consulting or advisory. Peter Vavassis reports a relationship with TerSera that includes board membership and consulting or advisory. Peter Vavassis reports a relationship with Tolmar that includes board membership, consulting or advisory, and speaking and lecture fees. The other authors declare that they have no known competing financial interests or personal relationships that could have appeared to influence the work reported in this paper.
